# Willingness to Use Digital Health Screening and Tracking Tools for Public Health in Sexual Minority Populations in a National Probability Sample: Quantitative Intersectional Analysis

**DOI:** 10.2196/47448

**Published:** 2024-03-08

**Authors:** Wilson Vincent

**Affiliations:** 1 Department of Psychology and Neuroscience, Temple University Philadelphia, PA United States

**Keywords:** COVID-19, demographics, digital health, disparities, intersectionality, mHealth, mobile health, psychometric properties, sexual minority populations

## Abstract

**Background:**

Little is known about sexual minority adults’ willingness to use digital health tools, such as pandemic-related tools for screening and tracking, outside of HIV prevention and intervention efforts for sexual minority men, specifically. Additionally, given the current cultural climate in the United States, heterosexual and sexual minority adults may differ in their willingness to use digital health tools, and there may be within-group differences among sexual minority adults.

**Objective:**

This study compared sexual minority and heterosexual adults’ willingness to use COVID-19–related digital health tools for public health screening and tracking and tested whether sexual minority adults differed from each other by age group, gender, and race or ethnicity.

**Methods:**

We analyzed data from a cross-sectional, national probability survey (n=2047) implemented from May 30 to June 8, 2020, in the United States during the height of the public health response to the COVID-19 pandemic. Using latent-variable modeling, heterosexual and sexual minority adults were tested for differences in their willingness to use digital health tools for public health screening and tracking. Among sexual minority adults, specifically, associations with age, gender, and race or ethnicity were assessed.

**Results:**

On average, sexual minority adults showed greater willingness to use digital health tools for screening and tracking than heterosexual adults (latent factor mean difference 0.46, 95% CI 0.15-0.77). Among sexual minority adults, there were no differences by age group, gender, or race or ethnicity. However, African American (*b*=0.41, 95% CI 0.19-0.62), Hispanic or Latino (*b*=0.36, 95% CI 0.18-0.55), and other racial or ethnic minority (*b*=0.54, 95% CI 0.31-0.77) heterosexual adults showed greater willingness to use digital health tools for screening and tracking than White heterosexual adults.

**Conclusions:**

In the United States, sexual minority adults were more willing to use digital health tools for screening and tracking than heterosexual adults. Sexual minority adults did not differ from each other by age, gender, or race or ethnicity in terms of their willingness to use these digital health tools, so no sexual orientation-based or intersectional disparities were identified. Furthermore, White heterosexual adults were less willing to use these tools than racial or ethnic minority heterosexual adults. Findings support the use of digital health tools with sexual minority adults, which could be important for other public health-related concerns (eg, the recent example of mpox). Additional studies are needed regarding the decision-making process of White heterosexual adults regarding the use of digital health tools to address public health crises, including pandemics or outbreaks that disproportionately affect minoritized populations.

## Introduction

Despite the economic, health, and mortality impacts of COVID-19 on the population as a whole, there has been great variability in the public’s willingness to participate in public health efforts to address the pandemic, including wearing masks and getting vaccinated [1,2]. Additionally, the COVID-19 pandemic has had an especially devastating impact on specific groups in the United States, with disproportionate mortality affecting older adults, men, and African American, and Hispanic American individuals [3-5]. Studies have examined the willingness to engage in preventive behaviors of American adults by age, gender, and race or ethnicity to curtail the COVID-19 pandemic, including the use of digital health tools for screening and tracking [6-11]. Although the COVID-19 pandemic has had a significant impact on sexual minority populations (ie, people who identify as gay, lesbian, bisexual, or other nonheterosexual sexual orientation identities) [12-14], less is known about sexual minority populations’ preventive responses to the COVID-19 pandemic. In the current climate of increased medical mistrust, the corresponding unwillingness to follow public health recommendations often occurs along demographic lines [15-17].

Some public health efforts for screening and tracking COVID-19, such as mobile health (mHealth), which includes the use of smartphones and related digital technologies to assess or address health, and other digital health tools (eg, patient portals and web-based patient questionnaires), have previously proven effective, acceptable, and feasible for HIV prevention for sexual minority men [18-21]. Generally, research shows that digital technologies such as mHealth applications improve the feasibility of delivering health care to sexual minority patients [22]. Additionally, studies indicate that tracking mental and physical health-related information through mHealth applications was associated with better mental health status for sexual minority people to a greater extent during the COVID-19 pandemic than before the pandemic [23]. Despite the potential for significant benefits, research has yet to examine sexual minority populations’ willingness to use mHealth tools for COVID-19.

For COVID-19, mobile apps created specifically in response to the COVID-19 pandemic have been the most frequently used type of mHealth tool [24]. Such mHealth tools aid in the screening, monitoring, and treatment of COVID-19 [24]. These mHealth-based approaches have had the advantage of easing the burden of in-person activities on COVID-19 testing and public health infrastructure (eg, avoiding supply chain issues, limiting the possibility of viral exposure) [18,25]. Previous research is mixed on whether there are no demographic differences by age, gender, or race or ethnicity in willingness to use digital health tools for COVID-19 screening and tracking. Although some studies showed no differences [26-28], others found evidence of greater willingness or support among younger adults, women, and racial and ethnic minority people [29-31]. However, these studies did not examine sexual orientation diversity. Heterosexual and sexual minority adults in the United States may differ in their willingness to participate in pandemic-related mHealth approaches, and these differences may vary based on other demographic characteristics such as age, gender, and race or ethnicity.

In addition to potential differences between heterosexual and sexual orientation–diverse populations, there may be notable differences among sexual minority populations. For example, the intersectionality theory asserts that seemingly independent yet intersecting social identities along social hierarchies based on dimensions such as race or ethnicity, gender, and sexual orientation jointly shape human experiences [32-35]. The intersectionality framework suggests that sexual minority people’s multiple intersecting identities must be considered simultaneously (eg, sexual minority people who are also African American), rather than treating each identity as a mutually exclusive category [36,37]. A recent review [38] and commentaries [39,40] have emphasized the urgent need to use intersectionality theory in the conceptualization and methodology of examining digital health disparities.

Although research has yet to explore sexual minority people’s willingness to use mHealth tools for COVID-19 based on their intersecting identities, such as age, gender, or race or ethnicity, the sexual minority health and HIV literatures illustrate how minoritized identities that intersect with sexual orientation–minoritized identities change the social position of individuals in ways that increase their risk of oppression and resulting adverse health outcomes. For example, Black sexual minority men experience both sexual orientation– and race-based stigma, and they experience more race- or ethnicity-based stigma in gay spaces than other groups [41,42]. Also, although both Black and White sexual minority men experience stereotypes about their sexual behaviors, assumptions may be more extreme for Black sexual minority men given the added layer of stereotypes about Black male sexuality [43,44]. The confluence of racism and antigay attitudes contributes to the increased risk of HIV for Black sexual minority men, including through social marginalization within communities of sexual minority men and late detection of HIV by medical and public health establishments, despite these men having no greater frequency or extent of sexual risk behavior to explain elevated HIV risk [42,45,46]. Additionally, Black sexual minority men may not benefit from or see the usefulness of mHealth tools for HIV prevention given that these tools may inadvertently stigmatize them through their “targeted” sexual health messages [47], or the tools may be viewed as a subpar offering in place of clinicians and public health professions “doing their jobs” [48]. Thus, although mHealth apps for HIV prevention have been found to be acceptable and feasible for implementation for sexual minority men in general [18-21], results may vary depending on intersecting racial, ethnic, or other identities.

This study examined the extent to which heterosexual adults and sexual minority adults differed in their willingness to participate in public health digital screening and tracking efforts to address the COVID-19 pandemic in a nationally representative sample of adults living in the United States. Also, for sexual minority participants, the author assessed differences within sexual minority populations in mean levels of willingness for COVID-19–related digital screening and tracking based on age group, gender, and race or ethnicity. Thus, the focus was on whether a sexual orientation–based disparity adversely affected sexual minority adults’ willingness to use digital health tools for screening and tracking and whether there were also intersectional disparities based on age, gender, and racial or ethnic categories.

## Methods

### Overview

This study was conducted and reported in accordance with the “Strengthening the Reporting of Observational Studies in Epidemiology” (STROBE) guidelines [49,50]. Specific study methods are provided in subsequent sections.

### Data

The COVID Impact Survey (CIS) is a national probability survey of US households designed to provide estimates for preventative behaviors and the impact of the COVID-19 pandemic; the data are publicly available [51]. The author used data from the last of 3 waves of cross-sectional data collection in the CIS, which occurred from May 30 to June 8, 2020 (n=2047). All 3 waves occurred between April 20 and June 8, 2020. These data were collected using the AmeriSpeak Panel, a probability-based panel distributed by NORC (formerly the National Opinion Research Center) at the University of Chicago.

US households were sampled with a known, nonzero probability of selection based on the NORC National Sample Frame, which was extracted from the US Postal Service Delivery Sequence File. Households were contacted by US mail, email, telephone, and field interviewers. The data are representative of noninstitutionalized adults who reside in the United States when weighted using sampling weights provider by the CIS. The CIS was funded by the Data Foundation. The NORC Institutional Review Board approved the CIS protocol to protect human participants (FWA00000142).

### Measures

#### Willingness for Public Health Digital Screening and Tracking for COVID-19

Participants responded to questions asking about their likelihood of COVID-19–related testing (ie, “Testing you for COVID-19 infection using a Q-tip to swab your cheek or nose” and “Testing you for immunity or resistance to COVID-19 by drawing a small amount of blood”) and digital screening and tracking (eg, “Installing an app on your phone that asks you questions about your own symptoms and provides recommendations about COVID-19” and “Installing an app on your phone that tracks your location and sends push notifications if you might have been exposed to COVID-19”). Response options ranged from (1) “extremely likely” to (5) “not at all likely.” Items were reverse-coded such that higher scores reflected a greater perceived likelihood for screening and tracking. Participants had the option to respond with (88) “Already done this,” and these cases were excluded using listwise deletion.

In a sample that included mostly heterosexual participants from Wave 2 of the CIS (manuscript under review), the measure showed construct validity in its positive correlations with participants having engaged in other protective behaviors to prevent COVID-19 infection (eg, “worn a face mask” and “avoided public or crowded places”). Additionally, participants who engaged in more frequent digital communications with friends and family before the public health response to the COVID-19 pandemic in the United States in March 2020 scored higher in willingness to use pandemic-related mHealth tools than participants who used digital communications with friends and family less frequently. The measure also showed measurement invariance across age groups, genders, and categories of race or ethnicity based on Wave 3. Based on Wave 1 of the CIS, the measure has demonstrated high internal consistency (Cronbach α=.90).

#### Demographic Characteristics

Participants self-reported their sexual orientation identity (ie, gay, lesbian, or bisexual, straight, something else, and I don’t know). Sexual orientation identity was dichotomized to reflect heterosexual status and nonheterosexual sexual-minority status, respectively. The following additional demographic characteristics were assessed for measurement invariance: age, gender, and race or ethnicity. Additionally, participants reported their current age, which the CIS categorized (ie, 18-24 years, 25-34 years, 45-54 years, 55-64 years, 65-74 years, and ≥75 years) to help anonymize the data set; gender (female coded 1, male coded 0); and self-identified race or ethnicity (eg, Black or African American, Hispanic or Latino, White, multiple other races and ethnicities, such as Asian, Indian, and Native Hawaiian). Transgender and nonbinary identities were not options on the CIS.

#### Data Analysis Plan

This study tested the extent to which heterosexual and sexual minority adults differed in their willingness to use digital health tools for public health screening and tracking, a latent variable, and whether sexual minority adults’ willingness to use these COVID-19–related digital health tools was associated with age, gender, and race or ethnicity. Measurement invariance (ie, whether the measure means and assesses the same thing across groups) was tested across heterosexual and sexual minority adults.

Descriptive statistics and Cronbach α were computed using Stata (version 16; StataCorp) [52], and coefficient ω and all analyses of associations and latent variables were conducted using M*plus* (version 8; Muthén & Muthén) [53]. Weighted least squares estimation with Delta parameterization was used to estimate model parameters [53]. This estimation method uses a diagonal weight matrix with SEs and mean- and variance-adjusted chi-square test statistics that rely on a full weight matrix (ie, ESTIMATOR=WLSMV in M*plus*) [53]. It is particularly appropriate for ordinal and nominal data [53]. Model fit was assessed with several fit indices based on any 2 of the following 3 criteria: a root-mean-square standard error of approximation (RMSEA) value of 0.06, a comparative fit index (CFI) value of at least 0.95, and a standardized root-mean-square residual (SRMR) criterion of 0.08 or less [54,55].

The author tested the extent to which the 5-item measure was invariant across the 2 sexual orientation categories. The 3 levels of measurement invariance—configural, metric, and scalar—were tested to determine if the 5-item measure was invariant across sexual-orientation categories. For ordinal variables and weighted least squares estimation methods with Delta parameterization, configural invariance (ie, pattern invariance), the least strict form of invariance, shows that each group has the same indicators loading onto the same factors in the same direction (ie, positive versus negative). To model configural invariance: (1) factor loadings are free to vary across groups, (2) thresholds are free to vary across groups, (3) scale factors are fixed to 1 in all groups, (4) factor means are fixed to 0 for all groups, and (5) factor variances are free to vary across groups [53]. Metric invariance (ie, weak invariance) indicates the invariance of factor loadings across groups, wherein (1) factor loadings are constrained to be equal across groups, (2) the first threshold of each item is constrained to be equal across groups, (3) the second threshold of the item that sets the metric of the factor is constrained to be equal across groups, (4) scale factors are fixed to 1 in 1 group and free to vary in the other groups, (5) factor means a Muthén & Muthénre fixed to 0 in 1 group and free to vary in the other groups, and (6) factor variances remain free to vary across groups [53]. Scalar invariance (ie, strong invariance) indicates equivalence of item intercepts or thresholds, in the case of categorical or ordinal variables, across groups and is the minimum needed to proceed with using a measure to test for differences in latent factor means between groups [56,57]. Scalar invariance is the same as metric invariance, except that thresholds are constrained to be equal across groups [53].

To compare invariance models, the author used a difference in CFI (ΔCFI) equal to or greater than 0.01 to indicate noninvariance [56]. Thus, a lack of worsened model fit with increased constraints indicates measurement invariance. Although scaled chi-square difference tests scaled for the weighted least squares estimator were conducted, this test may detect small discrepancies in ways that are not practically or theoretically meaningful in sample sizes greater than 200 [56-58].

Upon determining measurement invariance, the latent factor mean difference between heterosexual adults and sexual minority adults in the underlying factor of willingness to use digital health tools for public health screening and tracking was tested. Specifically, the latent variable for willingness to use digital health tools was standardized such that its mean was fixed to 0 and SD set to 1. The factor mean remained 0 for the reference group, heterosexual individuals, but the factor mean was freely estimated for the comparison group, sexual minority individuals. Thus, the resulting mean for sexual minority individuals reflected the difference in the mean from the reference group on a standardized metric, or in SD units. To identify correlates of willingness to use digital health tools for public health screening and tracking among sexual minority populations, specifically, willingness to use digital health tools was regressed on sexual minority adults’ age group, gender, and race or ethnicity, respectively.

Within an intersectionality-informed analytic framework, as described by Jackson et al [59], we can use additive measures of interaction to test for joint, referent, or excess intersectional disparities. Using the present analyses as a guiding example, the outcome variable would be recoded such that higher scores reflect a more adverse or disparity-oriented outcome (ie, less willingness to use COVID-19 screening and tracking tools). The predictor, gender (women coded 1), and the moderator, sexual orientation identity (sexual minority identity coded 1), would be coded such that the reference category (coded 0) is the nonminoritized group in this instance (ie, men and heterosexual adults) and the active category (coded 1) is the minoritized group (ie, women and sexual minority adults). Thus, the code of 1 reflects an adverse social position. For gender and sexual orientation, the original equation in the primary analyses before recoding and not including other covariates would be:


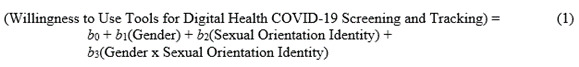


Given that the outcome should reflect a negative outcome to identify a disparity, the analyses would be repeated with the outcome variable recoded to reflect an unwillingness to use digital health tools rather than a willingness to use these tools. For gender and sexual orientation, the equation after recoding and not including other covariates would then be:


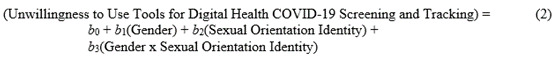


The joint disparity compares outcomes from the cell or group at the intersection of 2 minoritized identities, in this case, sexual minority women, to the group at the intersection of the 2 corresponding nonminoritized identities, in this instance, heterosexual men. In our example, *b*_1_ + *b*_2_ + *b*_3_ equals the joint disparity in unwillingness to use COVID-19 screening and tracking tools comparing sexual minority women to heterosexual men. Referent disparities are those that affect only 1 minoritized population or identity, in this case, women compared with men among heterosexual adults or heterosexual adults compared with sexual minority adults among men. It describes the disparity based on gender as a proxy for sexism or sexual minority identity as a proxy for heterosexism or homonegativity, but not both. Specifically, *b*_1_ equals the referent gender disparity in unwillingness to use COVID-19 digital screening among heterosexual adults, and *b*_2_ equals the referent sexual minority disparity among men. Finally, the excess intersectional disparity focuses on the intersection of minoritized identities and describes the extent to which the joint disparity exceeds the 2 individual referent disparities. Suppose it is greater than 0, or statistically significant. In that case, the strength of the association indicates the disparity at the intersection of minoritized gender and sexual orientation, that is, women who are also sexual minority adults, and *b*_3_ equals this excess intersectional disparity. A more detailed explanation can be found in Jackson et al [59] and VanderWeele and Tchetgen Tchetgen [60].

Disparities are indicated if the regression coefficients are positive, reflecting direct associations (ie, disadvantages for the minoritized groups) as opposed to inverse associations (ie, advantages for the more minoritized group). An advantage on an outcome for a relatively disadvantaged group that otherwise disproportionately and systematically experiences worse health outcomes and greater health risks would not meet established definitions of a disparity [61,62].

Given the complex nature of these survey data, analyses were adjusted using a sampling weight based on the inverse of the probability of selection in the sample. These analyses also accounted for stratification using pseudostrata based on census tracts. The data producer, NORC, used pseudostrata to preserve confidentiality. Per NORC, they did not include cluster variables because there were negligible cluster effects, and excluding these variables better preserved confidentiality (personal communication; Jennifer Benz, May 14, 2021). Descriptive statistics for the present sample accounted for weighting and stratification to reflect the complex survey design and national representativeness of the sample along key raking variables (ie, age, gender, and race or ethnicity). Latent factor mean differences (ΔM) and regression coefficients (*b*) are presented with their 95% CIs. Missing data, which were up to 3.7% missing across analyses, were handled using listwise deletion.

### Ethical Considerations

Temple University’s institutional review board determined that the present analyses, which used deidentified publicly available data, did not require institutional approval for human participants research (contact the corresponding author for documentation).

## Results

### Sample Characteristics

Of the total sample of 1928 adults, 161 were sexual minority individuals. Other sample characteristics are listed in Tables 1 and 2. The sample size was reduced from 2047 due to missing data on sexual orientation (6.2%).

**Table 1:** Descriptive statistics based on raking variables (ie, age, gender, and race or ethnicity) given complex design and weighting and sexual orientation identities represented in the sample (N=1928). Proportions may not sum to 1, and percentages may not sum to 100 due to rounding. Subcategories (eg, sexual orientation subcategories) may not sum to 1928 due to missing data. Sexual orientation was significantly associated with age group; the pattern of results indicates that younger adults were more likely to self-identify as sexual minority individuals than older adults.

Variable		Total					Estimated proportion			Comparison linearized SE		*P* value	
		n	Estimated proportion (%)	Linearized SE	Design effect	Heterosexual		Sexual minority					
**Age (years)**													<.001
	18-29	311	0.211	0.015	2.48	0.882		0.118	0.025				
	30-44	594	0.263	0.013	1.62	0.916		0.085	0.015				
	45-59	435	0.238	0.013	1.74	0.945		0.055	0.012				
	≥60	588	0.288	0.013	1.675	0.966		0.034	0.008				
**Gender**													.19
	Male	914	0.478	0.015	1.82	0.940		0.060	0.009				
	Female	1014	0.522	0.015	1.82	0.921		0.079	0.012				
**Race or ethnicity**													.72
	Asian or Asian American	62	0.056	0.008	2.47	0.956		0.044	0.027				
	Black or African American	227	0.124	0.010	1.83	0.920		0.080	0.026				
	Hispanic or Latino	324	0.166	0.012	2.01	0.919		0.081	0.019				
	White or European American	1127	0.623	0.015	1.84	0.934		0.066	0.010				
	Other races and ethnicities	103	0.031	0.004	0.892	0.906		0.095	0.029				
**Sexual orientation**													—^a^
	Homosexual	58	3.0	—	—	—		—	—				
	Bisexual	69	3.6	—	—	—		—	—				
	Heterosexual	1767	91.7	—	—	—		—	—				
	Other: “Something else”	25	1.3	—	—	—		—	—				
	Other: “I don’t know the answer”	9	0.5	—	—	—		—	—				

^a^Not available.

**Table 2:** Descriptive statistics based on sexual orientation and willingness to use mHealth tools for pandemic-related digital screening and tracking (“If these options were available to you, how likely would you be to participate in them?”; N=1928). Proportions may not sum to 1, and percentages may not sum to 100 due to rounding. Subcategories (eg, sexual orientation subcategories) may not sum to 1928 due to missing data. Sexual orientation was significantly associated with willingness to install “an app on your phone that tracks your location and sends push notifications if you might have been exposed to COVID-19”; the pattern of results indicates that sexual minority adults reported being more likely to install such an app than heterosexual adults. Additionally, sexual orientation was also significantly associated with willingness to “use a website to log your symptoms and location and get recommendations about COVID-19”; the pattern of findings indicates that sexual minority adults reported being more likely to use such a website than heterosexual adults.

Variable			Total									Estimated proportion					Comparison linearized SE			*P* value	
			N		Estimated proportion (%)		Linearized SE		Design effect		Heterosexual			Sexual minority							
**Installing an app on your phone that asks you questions about your own symptoms and provides recommendations about COVID-19**																					.06
	Extremely likely	146		0.075		0.008		1.75		0.866			0.134		0.044						
	Very likely	212		0.110		0.010		1.95		0.898			0.102		0.028						
	Moderately likely	373		0.203		0.013		2.04		0.927			0.073		0.018						
	Not too likely	404		0.219		0.013		1.85		0.937			0.063		0.014						
	Not likely at all	757		0.377		0.015		1.73		0.950			0.050		0.009						
	Already done this	27		0.017		0.004		1.89		0.893			0.107		0.062						
**Installing an app on your phone that tracks your location and sends push notifications if you might have been exposed to COVID-19**																					<.001
	Extremely likely	171		0.086		0.008		1.69		0.826			0.174		0.045						
	Very likely	232		0.130		0.011		1.88		0.889			0.111		0.029						
	Moderately likely	365		0.202		0.013		1.95		0.947			0.053		0.011						
	Not too likely	342		0.180		0.013		2.17		0.958			0.042		0.012						
	Not likely at all	790		0.394		0.015		1.71		0.943			0.057		0.010						
	Already done this	15		0.007		0.002		1.54		0.948			0.052		0.052						
**Using a website to log your symptoms and location and get recommendations about COVID-19**																					.008
	Extremely likely	129		0.074		0.009		2.40		0.842			0.158		0.048						
	Very likely	216		0.112		0.009		1.72		0.906			0.095		0.025						
	Moderately likely	441		0.240		0.014		2.00		0.917			0.083		0.016						
	Not too likely	403		0.204		0.012		1.72		0.953			0.047		0.014						
	Not likely at all	710		0.631		0.012		1.73		0.949			0.051		0.010						
	Already done this	15		0.009		0.003		1.81		1.00			0.000		0.000						

### Psychometric Properties of Measure of Willingness to Use Digital Health Tools for COVID-19–Related Screening and Tracking

The measure of willingness to use digital health tools for COVID-19–related screening and tracking showed internal consistency and reliability (Cronbach α=.89 and coefficient ω=0.93). Additionally, as shown in Table 3, the measure was invariant by sexual orientation. Configural invariance was indicated by all factor loadings being significant and in the expected direction for each group. The configural model had no global fit statistics, as it was a fully saturated model. Next, the author tested a metric invariance model with factor loadings constrained to be equal across groups, and metric invariance was evident (ΔCFI<0.01; Δ*χ*^2^_2_=2.30; *P*=.32). Thus, the metric model had an equivalent model fit with the configural invariance model; the nonsaturated metric model fit the data, per the RMSEA, CFI, and SRMR (Table 3). Finally, the author tested a scalar invariance model, and scalar invariance was shown (ΔCFI<0.01; Δ*χ*^2^_8_=6.44; *P*=.60).

**Table 3:** Measurement invariance by sexual orientation for a measure of pandemic-related psychological distress (N=1928). Sexual orientation categories are heterosexual and sexual minority. The measure showed measurement in variance based on the criterion of ΔCFI<.01 [54] between the configural and the metric model and between the metric and the scalar model.

		Fit index				
		Chi-square (*df*)	*P* value	RMSEA^a^	CFI^b^	SRMR^c^
Invariance models						
	Configural	0.00 (0)	<.001	0.00	1.00	0.00
	Metric	2.30 (2)	.32	0.01	1.00	<0.01
	Scalar	8.51 (10)	.58	<0.01	1.00	0.01

^a^RMSEA: root-mean-square standard error of approximation.

^b^CFI: comparative fit index.

^c^SRMR: standardized root-mean-square residual.

### Mean Difference by Sexual Orientation on Willingness to Use Digital Health Tools for COVID-19–Related Screening and Tracking

Given scalar invariance, factor means for willingness to use COVID-19–related digital screening and tracking tools differed between heterosexual and sexual minority adults. Specifically, willingness to use digital health tools was nearly half an SD greater for sexual minority adults than for heterosexual adults (ΔM=0.46, 95% CI 0.15-0.77).

### Associations Between Demographic Characteristics and Willingness to Use Digital Health Tools for COVID-19–Related Screening and Tracking Among Sexual Minority Adults

Within the population of sexual minority adults, no differences were detected by age group, gender, or race or ethnicity in their willingness to use digital health tools for COVID-19–related screening and tracking. Specifically, as detailed in Table 4, for each increase in age by group, there was no change in willingness to use digital health tools. Also, men and women did not differ in their willingness to use digital health tools. Finally, African American individuals, Hispanic or Latino individuals, and people of other races or ethnicities did not differ from White individuals (the reference group) in their willingness to use digital health tools.

**Table 4:** The independent associations of age, gender, and race and ethnicity with willingness to use digital health tools for public health screening and tracking (latent variable) in separate structural equation models in a national probability sample of sexual minority people (N=161).

Variable				Structural equation models										
				*b* ^a^		95% CI		*R* ^2b^		RMSEA^c^		CFI^d^		SRMR^e^
**Age (years)**														
	Age group^f^			0.04		–0.12 to 0.21		0.01		0.17		0.99		0.04
**Gender**														
	Female (reference: male)			–0.14		–0.66 to 0.38		0.01		0.00		1.00		0.02
**Race or ethnicity**														
	**Race or ethnicity (reference: White or European American)**							0.01		0.00		1.00		0.03
		Black or African American	0.03		–0.69 to 0.75									
		Hispanic or Latino	0.09		–0.45 to 0.64									
		Other race or ethnicity	–0.20		–1.02 to 0.62									

^a^*b*=unstandardized regression coefficient.

^b^*R*^2^=coefficient of determination.

^c^RMSEA: root-mean-square standard error of approximation.

^d^CFI: comparative fit index.

^e^SRMR: standardized root-mean-square residual.

^f^Age group is an ordinal variable with levels as follows: (1) 18-24 years, (2) 25-34 years, (3) 35-44 years, (4) 45-54 years, (5) 55-64 years, (6) 65-74 years, and (7) ≥75 years.

Additional models were tested for interactions of sexual minority status as a moderator with the other demographic characteristics as respective predictors in their associations with willingness to use digital health tools for screening and tracking. None of the interaction terms reached statistical significance in the models (Table 5). However, with the inclusion of the interaction terms, the main effects of being African American, Hispanic or Latino, and another racial or ethnic identity reached statistical significance. Specifically, among heterosexual adults, being Black or African American was associated with 41% of an SD greater willingness to use digital health screening and tracking tools (*b*=0.41, CI 0.19-0.62), being Hispanic or Latino was associated with 36% of an SD greater willingness to use these tools (*b*=0.36, CI 0.18-0.55), and being of another racial or ethnic minority group was associated with 54% of an SD greater willingness to use these tools (*b*=0.54, CI 0.31-0.77).

**Table 5:** The independent associations of age, gender, and race or ethnicity and their respective interactions by sexual minority status with willingness to use digital health tools for public health screening and tracking (latent variable) in separate structural equation models in a national probability sample of sexual minority people (N=1928).

Variable				Structural equation models														
			*b* ^a^		95% CI		*R^2^^b^*			RMSEA^c^			CFI^d^			SRMR^e^		
**Age (years)**									0.02			0.03			1.00			0.01
	Age group^f^		–0.02		–0.06 to 0.02													
	Sexual minority (reference: heterosexual)		0.28		–0.32 to 0.88													
	Age group × sexual minority		0.06		–0.10 to 0.22													
**Gender**									0.02			0.00			1.00			0.01
	Female (reference: male)		0.05		–0.08 to 0.19													
	Sexual minority		0.58		0.25 to 0.91													
	Female × sexual minority		–0.19		–0.68 to 0.31													
**Race or ethnicity**																		
	**Race or ethnicity (reference: White or European American)**							0.05			0.01			1.00			0.01	
		Black or African American	0.41		0.19 to 0.62													
		Hispanic or Latino	0.36		0.18 to 0.55													
		Other race or ethnicity	0.54		0.31 to 0.77													
Sexual minority				0.61		0.28 to 0.94												
**Race or ethnicity × sexual minority**																		
	Black/African American × sexual minority		–0.35		–1.05 to 0.35													
	Hispanic or Latino × sexual minority		–0.21		–0.77 to 0.35													
	Other race or ethnicity × sexual minority		–0.75		–1.57 to 0.07													

^a^*b*=unstandardized regression coefficient.

^b^*R*^2^=coefficient of determination.

^c^RMSEA: root-mean-square standard error of approximation.

^d^CFI: comparative fit index.

^e^SRMR: standardized root-mean-square residual.

^f^Age group is an ordinal variable with levels as follows: (1) 18-24 years, (2) 25-34 years, (3) 35-44 years, (4) 45-54 years, (5) 55-64 years, (6) 65-74 years, and (7) ≥75 years.

The models were re-run with the outcome variable recoded to identify referent, excess intersectional, and joint disparities. Given the direction of the significant associations for each racial or ethnic minority group (Black or African American: *b*=–0.41, CI –0.62 to –0.19; Hispanic or Latino: *b*=–0.36, CI –0.55 to –0.18; and other racial or ethnic minority group: *b*=–0.54, CI –0.77 to –0.31), referent racial or ethnic or sexual orientation-based disparities were not detected. Also, no excess intersectional disparity was detected (Black or African American × sexual minority: *b*=–0.35, CI –0.35 to 1.05; Hispanic or Latino × sexual minority: *b*=0.21, CI –0.35 to 0.77; and other racial or ethnic minority group × sexual minority: *b*=–0.75, CI –0.07 to 1.57). Overall, no joint disparity was identified.

## Discussion

### Overview

Studies rarely examine the willingness of sexual minority populations to use mHealth and related digital health tools in the context of pandemic-related or non-HIV prevention. However, such mHealth tools have been acceptable and effective when used to fight the HIV epidemic for sexual minority men, specifically [18-21]. This study examined the use of digital health tools for screening and tracking for the COVID-19 pandemic, focusing on a broader, more diverse range of the sexual minority population in the United States. In particular, the study used the conceptual [32,33] and methodological [34,35,39] frameworks of intersectionality theory to determine the presence of disparities between heterosexual and sexual minority adults across various intersections of identity (ie, age, gender, and race or ethnicity) in their willingness to use digital health tools for screening and tracking.

Findings indicated that sexual minority adults were significantly more willing to use digital health tools for screening and tracking than heterosexual adults in the United States. The greater willingness to use digital health tools among sexual minority adults compared with heterosexual adults might be explained partly by the familiarity of many sexual minority individuals with the use of mHealth and other digital health methods for outreach and other public health efforts, particularly sexual minority men in the context of HIV prevention [18-21].

Despite the difference between heterosexual adults and sexual minority adults in this study, there were no within-group demographic differences among sexual minority adults in their willingness to use digital health tools for screening and tracking. These findings from the United States are consistent with findings from a large survey of registered National Health Service users in the United Kingdom, in which there were no differences by age or gender in terms of willingness to participate in contact tracing through a mobile phone app in the adult population as a whole [63]. Interestingly, although sexual minority men are often more likely to be the focus of mHealth interventions for HIV [20,21], they were no more likely than sexual minority women to express a willingness to use digital health tools for screening and tracking in this study.

Based on the established definitions of a disparity [61,62] and the analytic framework of intersectionality theory [32,33,59], this study detected no referent disparity based on sexual orientation and no joint or excess joint disparity at the intersection of sexual orientation identity and other demographic characteristics. A previous study that used the same publicly available data without testing for sexual minority status as a predictor or moderator found no significant associations that would indicate an age-related, gender, or racial or ethnic disparity [28]. In contrast to other studies, which showed mixed findings for race or ethnicity and other demographics as a predictor without considering sexual minority status [26,27,29-31], the significant main effects of race or ethnicity in this study occurred among heterosexual adults in the presence of interactions of race or ethnicity with sexual minority status.

The differences between racial or ethnic minority adults and White adults may be explained, in part, by political ideology affecting attitudes toward the public health establishment during COVID-19. For example, a study found that moderate- and conservative-leaning respondents showed less support for using COVID-19–related digital health tools than liberal-leaning respondents in the same model in which racial and ethnic minorities showed greater support for using COVID-19–related digital health tools than White Americans or non-Hispanic Americans [31]. Additionally, studies indicate that some White Americans may be increasingly voting conservative [64-66]. To the extent that these political ideologies are also tied to public health mistrust, there may be noteworthy consequences. For example, there is evidence of excess deaths for conservative-voting adults compared with less conservative-voting adults in Florida and Ohio during the COVID-19 pandemic [67]. The CIS did not include questions regarding political beliefs. As such, this study did not test whether demographic factors interacted with political ideology, which would have helped to determine whether political ideology mattered within each racial or ethnic category. Additional studies are needed to examine the decision-making process of White heterosexual adults regarding their use of digital health tools for screening and tracking during public health emergencies.

The present psychometric evaluation indicated that the COVID-19–related psychological distress measure was assessing the same construct in heterosexual participants and sexual minority participants. A previous study has already validated the psychometric properties of the present items (eg, construct validity and internal consistency) and demonstrated measurement invariance across age groups, genders, and races and ethnicities [28]. Other studies on willingness to use digital health tools have typically used a single item [63] or several items treated as separate measures [68,69] rather than a single, validated scale. In terms of scales that use any variation on willingness (eg, intentions and perceived usefulness), 1 study used a 15-item measure [70] and another study used 2 measures of 32 items each [71]; these are notably longer than the 3-item measure of this study. Some studies have used items with binary yes-or-no responses [63,68,69], which may not capture sufficient gradation in response if the goal is to understand the degree of willingness.

### Strengths and Limitations

This study has multiple strengths. For example, the study used a national probability sample to represent the population of noninstitutionalized adults in the United States. Additionally, the study used innovative methods to conceptualize and quantitatively identify disparities within the framework of intersectionality theory. In addition, the study established measurement invariance between heterosexual and sexual orientation–diverse adults for a measure of willingness to use a digital health screening and tracking tool that was previously validated by age, gender, and race or ethnicity. The measure can be adapted for screening and tracking in response to future public health events.

Additionally, several limitations must be noted. Specifically, the cross-sectional study design precludes definitive causal conclusions. In addition, the author did not attempt to draw conclusions about the temporal associations among the variables. Moreover, the sample was imbalanced with respect to the proportion of sexual minority adults compared with heterosexual adults in the sample; the number of sexual minority adults was much smaller. Limitations also include a lack of questions measuring sexual and gender identity that follow best practice [72], including the lack of transgender-inclusive gender questions and the lack of sexual orientation measures that distinguish different sexual orientation groups beyond sexual minority status by gender (ie, sexual minority men, including gay and bisexual men, and women, including lesbians and bisexual women, which were accounted for in this study). As a result, the analyses were not more nuanced regarding gender and sexual orientation identity.

### Implications

This study has several research and applied implications. For instance, additional research can oversample sexual minority adults to provide balanced samples for comparisons between heterosexual and sexual minority adults. Additionally, studies can examine sexual minority individuals’ willingness to use digital health tools for other non-COVID-19–related health issues beyond HIV, including specific mental health diagnoses (eg, depression and substance use) and chronic illnesses (eg, diabetes and hypertension). These studies should consider intersections of identities among sexual minority people, such as underrepresented racial and ethnic minority people among sexual minority populations. Recently, monkey pox has emerged among sexual minority men, in particular [73], and digital health approaches may be useful in such circumstances. Additionally, studies are needed to further examine the decision-making process of White heterosexual adults regarding their use of digital health tools in response to public health emergencies.

Regarding applied implications, public health professionals and clinicians should consider screening sexual minority adults for their willingness to use digital health tools as they continue to use telehealth during COVID-19 and post–COVID-19 times. Such screening is particularly needed for sexual minority adults who contend with intersecting systems of oppression and identities and, thus, have elevated levels of medical mistrust (eg, underrepresented ethnic and sexual minority people) [15,17,74]. Policy changes and other structural interventions are needed to provide access to digital health technologies in cases in which willingness to use these technologies does not appear to be the issue. In this study, racial and ethnic minority heterosexual adults seemed particularly willing to use these technologies.

### Conclusions

This study is responsive to recent calls in the literature to address the pronounced dearth of intersectionality theory-informed research investigating disparities related to digital health [38-40]. As we strive to narrow the digital divide, or the disparities in technology and internet access and use [75-77], we must understand disparities in willingness to use digital technologies for health-related purposes even when these tools are available. This study detected no disparities based on sexual minority status or intersections of identity among sexual minority adults along age, gender, or race or ethnicity. As such, for sexual minority populations, including intersections that are at joint or compound risk of experiencing adverse health outcomes, the issue is not willingness to use digital health tools compared to heterosexual adults. Sexual minority populations require culturally responsive digital health approaches to address their needs, as opposed to motivational enhancement or other interventions to increase their willingness during public health events. The willingness of sexual minority adults across intersecting identities to use pandemic-related digital health tools, including mobile health apps, is noteworthy given the potential promise of digital health tools for other public health-related concerns, such as the recently ended mpox outbreak [78,79], which disproportionately affected sexual minority men [80-82], and obesity [83,84] and cardiovascular disease [85,86], which disproportionately affects sexual minority women [87,88]. Additionally, White heterosexual adults demonstrated a disproportionately low willingness to use digital health tools, and this may become an issue in the event that this population is adversely affected by a public health concern that can benefit from digital health technologies.

## Abbreviations

CFI: comparative fit index
CIS: COVID Impact Survey
mHealth: mobile health
NORC: National Opinion Research Center
RMSEA: root-mean-square standard error of approximation
SRMR: standardized root-mean-square residual
STROBE: Strengthening the Reporting of Observational Studies in Epidemiology

## Data Availability

The data sets analyzed during this study are available in the NORC at the University of Chicago repository [51].
